# A recombinant technique for mapping functional sites of heterotrimeric collagen helices: Collagen IV CB3 fragment as a prototype for integrin binding

**DOI:** 10.1016/j.jbc.2023.104901

**Published:** 2023-06-10

**Authors:** Sergei P. Boudko, Elizabeth H. Konopka, Woojin Kim, Yuki Taga, Kazunori Mizuno, Timothy A. Springer, Billy G. Hudson, Terence I. Moy, Fu-Yang Lin

**Affiliations:** 1 Division of Nephrology and Hypertension, Department of Medicine, Vanderbilt University Medical Center, Nashville, Tennessee, USA; 2 Center for Matrix Biology, Vanderbilt University Medical Center, Nashville, Tennessee, USA; 3 Department of Biochemistry, Vanderbilt University, Nashville, Tennessee, USA; 4 Morphic Therapeutic, Inc, Waltham, Massachusetts, USA; 5 Nippi Research Institute of Biomatrix, Toride, Ibaraki, Japan; 6 Department of Biological Chemistry and Molecular Pharmacology, Program in Cellular and Molecular Medicine, Boston Children's Hospital, Harvard Medical School, Boston, Massachusetts, USA; 7 Department of Pathology, Microbiology, and Immunology, Vanderbilt University Medical Center, Nashville, Tennessee, USA; 8 Department of Cell and Developmental Biology, Vanderbilt University Medical Center, Nashville, Tennessee, USA; 9 Vanderbilt-Ingram Cancer Center, Vanderbilt University, Nashville, Tennessee, USA; 10 Vanderbilt Institute of Chemical Biology, Vanderbilt University, Nashville, Tennessee, USA

**Keywords:** collagen IV, integrin, basement membrane, receptor, triple helix, extracellular matrix, heterotrimer, CB3 fragment, cystine knot, protein self-assembly, recombinant protein expression, protein folding, atomic force microscopy, CD spectroscopy

## Abstract

Collagen superfamily of proteins is a major component of the extracellular matrix. Defects in collagens underlie the cause of nearly 40 human genetic diseases in millions of people worldwide. Pathogenesis typically involves genetic alterations of the triple helix, a hallmark structural feature that bestows exceptional mechanical resistance to tensile forces and a capacity to bind a plethora of macromolecules. Yet, there is a paramount knowledge gap in understanding the functionality of distinct sites along the triple helix. Here, we present a recombinant technique to produce triple helical fragments for functional studies. The experimental strategy utilizes the unique capacity of the NC2 heterotrimerization domain of collagen IX to drive three α-chain selection and registering the triple helix stagger. For proof of principle, we produced and characterized long triple helical fragments of collagen IV that were expressed in a mammalian system. The heterotrimeric fragments encompassed the CB3 trimeric peptide of collagen IV, which harbors the binding motifs for α_1_β_1_ and α_2_β_1_ integrins. Fragments were characterized and shown to have a stable triple helix, post-translational modifications, and high affinity and specific binding of integrins. The NC2 technique is a universal tool for the high-yield production of heterotrimeric fragments of collagens. Fragments are suitable for mapping functional sites, determining coding sequences of binding sites, elucidating pathogenicity and pathogenic mechanisms of genetic mutations, and production of fragments for protein replacement therapy.

Collagens are a major component of the extracellular matrix. They are comprised of 28 types in humans (I–XXVIII) encoded by over 40 different genes, forming a diversity of triple helical protomers of varying α-chain compositions (1, 2). Protomers assemble into diverse superstructures, ranging from networks to fibrils and broadly function in structural, mechanical, and organizational roles that define tissue architecture and influence cellular behavior. Defects in collagens underlie the cause of nearly 40 human genetic diseases, affecting numerous organs and tissues in millions of people worldwide (3). Pathogenesis typically involves genetic alterations of the triple helix, a hallmark structural feature that bestows exceptional mechanical resistance to tensile forces and a capacity to bind a plethora of macromolecules. Such macromolecules include but are not limited to integrins, DDR1 and 2, fibronectin, nidogen, perlecan, heparin, von Willebrand factor, decorin, bone morphogenetic proteins, and glycoprotein VI (2, 4, 5). Yet, there is a paramount knowledge gap in understanding the functionality of distinct sites along the triple helix. This lack of knowledge impedes the development of precision therapies aimed at restoring/repairing function of collagen superstructures.

A unique feature of the collagen triple helix is a register (or stagger) of chains (6). There are always leading, middle, and trailing chains shifted by one residue. Even in homotrimeric types of collagens, identical residues within the triple helix are not structurally equivalent. At least seven types of human collagens, that is, I, IV, V, VI, VIII, IX, and XI, exist as heterotrimers of either AAB or ABC forms (2), which represent an additional challenge in generating biologically relevant collagen fragments. Examples include collagen I, the most abundant fibrillar type with an α1α1α2 composition and collagen IV of basement membrane with three compositions in mammals—α1α1α2, α3α4α5, and α5α5α6 (7, 8).

Generation of short fragments of homotrimeric collagens using chemical synthesis became a general method, and great progress was achieved using this approach. Synthetic peptides were used to solve the first crystal structures of a collagen triple helix (9), the collagen triple helix with a mutation (10), and a collagen triple helix complex with integrin α_2_β_1_ (11). Peptide toolkits covering the entire triple-helical domains of homotrimeric collagens II and III were successfully implemented and used to precisely map and study binding to integrin α_2_β_1_ (12), von Willebrand factor (13), DDR1 (14), DDR2 (15), dermatopontin (16), HSP47 (17), gpVI (18), LAIR-1 (19), SPARC (20), MMP-3 (21), multimerin 1 (22), and other proteins (reviewed by Farndale (23)). Several strategies were developed to address production of heterotrimeric collagen fragments using regioselective chemical synthesis (24, 25, 26) or hosting heterotrimers with artificial sequence designs (27, 28) (reviewed by Xu and Kirchner (29)). However, these approaches are limited to relatively short fragments (up to 12 residues) and require sophisticated approaches of chemical synthesis. So far, no collagen peptide toolkits have been reported for any heterotrimeric type of collagen.

Here, we present a recombinant technique to produce heterotrimeric (triple helical) fragments for mapping functional sites, determining coding sequences of binding sites, elucidating pathogenicity and pathogenic mechanisms of genetic mutations, and production of fragments for protein replacement therapy. The experimental strategy utilizes the unique property of the noncollagenous (NC) 2 heterotrimerization domain of collagen IX. We previously demonstrated that the NC2 domain is sufficient to drive three α-chain selections and register the triple helix stagger using a bacterial system (30, 31, 32). For proof of principle, we adapted the NC2 domain for studies of collagen IV (Fig. 1), which harbors binding sites for numerous macromolecules (3, 4). Long triple helical fragments that encompass the CB3 site that harbors the binding motifs for α_1_β_1_ and α_2_β_1_ integrins (Fig. 1) were expressed and characterized. Fragments were shown to have a stable triple helix, post-translational modifications (PTMs), and specific binding to integrins.

**Figure 1:**
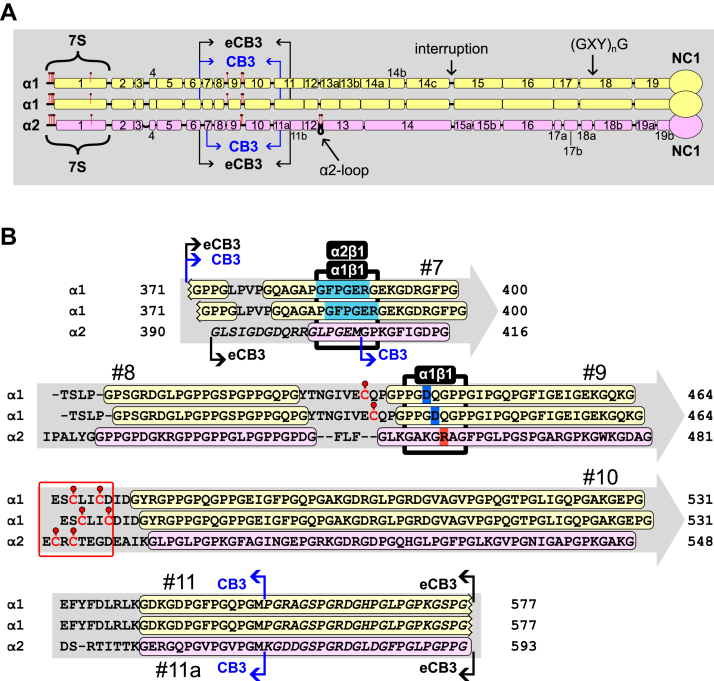
**Collagen IV and CB3-extended fragment used in this study.***A*, schematic drawing of primary sequences of collagen IV α1, α1, and α2 chains that assemble into the heterotrimeric protomer. Triple helical segments (GXY)_n_G are depicted as *colored bars* connected with *black lines* representing interruptions of various lengths. Predicted helical segments are numbered from N to C termini. Cysteines are depicted as *red pins*. *Blue arrows* mark the reported CB3 cleavage sites (33). *Black arrows* label positions of the extended CB3 (eCB3) sequences used in this study. *B*, the eCB3 sequences used to generate recombinant fragments in this study. *Colored bars* highlight triple helical segments. Most helical segments are shown at specific registers. Those registers are suggestive, and the one for helix #9 is in agreement with the reported one (39). *Black underlying boxes* highlight binding sites for α_1_β_1_ and α_2_β_1_ integrins. GFPGER sequence, the major α_2_β_1_ integrin–binding site and also the α_1_β_1_ integrin–binding site is highlighted with *cyan background*. The suggested chain-distributed recognition site for the α_1_β_1_ integrin is in addition highlighted for D (*blue background*) and R (*orange background*) residues. Cysteines are shown as *red pins*. *Red box* highlights sequences forming the CB3 cystine knot. The UniProt identifiers for sequences are P02462 for α1 chain (isoform 1) and P08572 for α2 chain.

## Results

### Design and production

**Figure 2:**
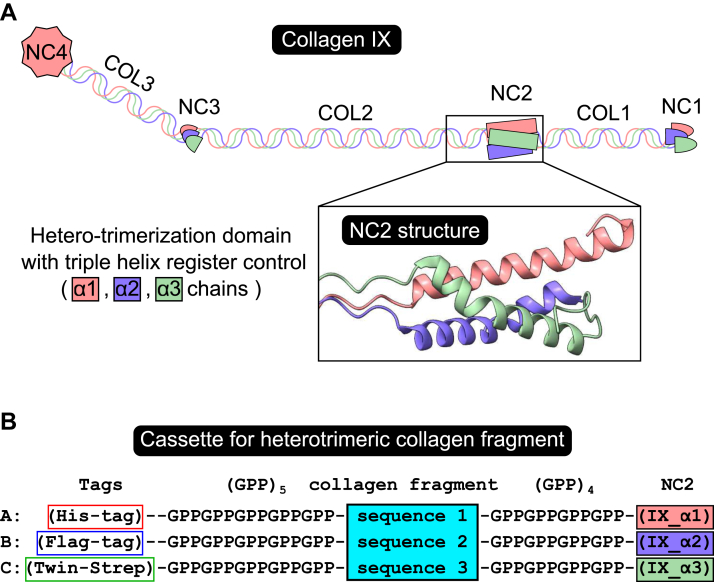
**A tool for producing heterotrimeric collagen fragments.***A*, collagen IX has three unique chains, α1, α2, and α3. The collagen IX assembly has three collagenous (COL1–COL4) and four noncollagenous (NC1–NC4) regions. The NC2 region is sufficient to select chains, trimerize them, and define the register of an adjacent triple helix (30, 31). It can be used to drive the assembly of homotrimeric and heterotrimeric fragments of other collagens. *B*, cassette of three chimeric sequences A, B, and C, where collagenous region of interest (sequences 1–3 corresponding to three chains of desired composition and register) flanked by collagen-like repeats ([GPP]_5_ and [GPP]_4_) is followed by the NC2 domain (chains α1–α3). Combination of all three chains results in the NC2 heterotrimer assembly, which initiates folding of the collagenous part. To facilitate purification and ensure robust heterotrimeric composition, each chain contains a unique tag.

**Figure 3:**
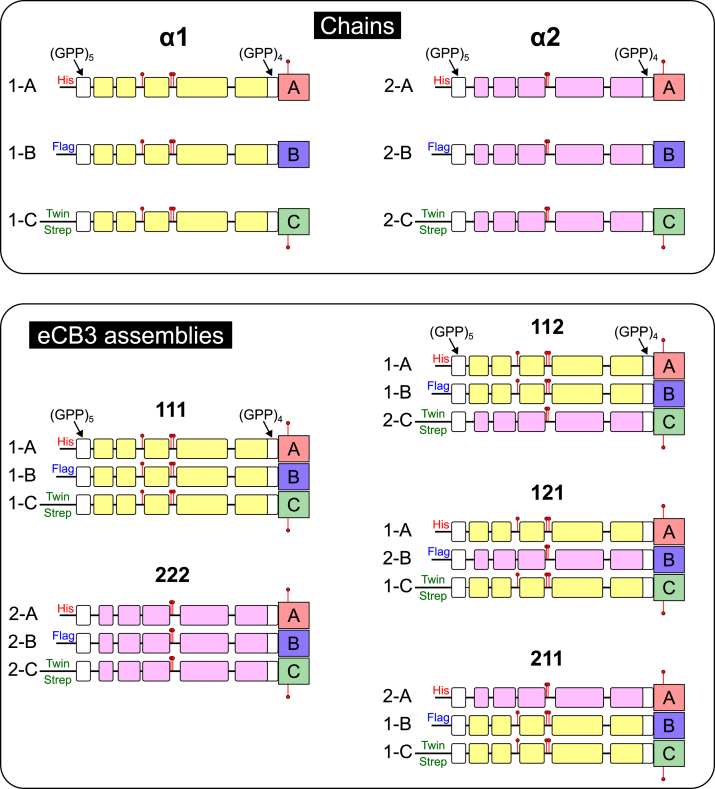
**The chains and eCB3 assemblies.** Schematic presentation of primary sequences of chains coexpressed to assemble 111, 222, 112, 121, and 211 eCB3 fragments. Linear scale corresponds to number of residues. The signal peptide sequences are excluded. Triple helical sequences are depicted as *bars*, *yellow color* corresponds to α1 chain of human collagen IV, *light purple* corresponds to α2, *white* to artificial GPP repeats. The sequences for NC2 domain are shown as *pink*, *light blue*, and *light green rectangles* and labeled A, B, and C, which corresponds to α1, α2, and α3 chains of human collagen IX. Interruptions, linkers, and tags are depicted as *black lines*. Cysteines are depicted as *red pins*.

Expression of artificial or fragmentary collagen triple helices that exhibit native structure and function has proven difficult as they are composed of two or three distinct α-chains. It has proven difficult to control the stoichiometry of the assembled collagen fragment. Moreover, collagen sequences require PTMs, that is, hydroxylation and glycosylation, in order to form a stable and functional triple helical structure. We thus sought an expression system in mammalian cells that would facilitate expression of collagen domains whose collagen subunit composition and helix register could be controlled. Previously, we demonstrated that three chains of the collagen IX NC2 domain, one for each subunit, are sufficient to drive subunit selection, that is, appropriate subunit composition and to control the register of the triple helical stagger (30, 31, 32). Here, we adapt this system to produce a long fragment of collagen IV (∼200 residues) in a mammalian expression system. We selected the CB3 region of human collagen IV as its most well-characterized fragment (33, 34, 35, 36, 37, 38, 39, 40, 41). The CB3 region of collagen IV was initially identified as a fragment chemically excised with cyanogen bromide (CNBr) that could be prepared in relatively high yield for structural and functional studies (33). Typical of collagen IV domains, CB3 has interruptions and a cystine knot as well as containing other known protein-binding sites (4). The cystine knot seems to be an evolutionary conserved structural element in collagen IV also found in such distant organisms as fruit flies, roundworms, and even in Cnidaria (Fig. S1). We generated expression constructs that extended the sequences of CB3 to a gelatinase A cleavage site at the N terminus (38) and completed the last GXY repeat in the α2 chain to ensure the continuity and stability of the triple helices (Figure 1, Figure 2, Figure 3 and S2). We refer to this genetically encoded CB3 domain as extended CB3 (eCB3).

As in the bacterial expression system (30, 32), for each chain (α1 and α2) of eCB3, we generated three constructs, each containing one of the three nonidentical NC2 trimerization domains of collagen IX, that is, α1 NC2, α2 NC2, and α3 NC2. These domains pack in a specific orientation around the threefold pseudosymmetry axis of collagen and define the stagger of the collagen triple helices (31). Each of the three eCB3 domain sequences was cloned upstream of its specific NC2 domain and flanked by GPP repeats to avoid destabilizing effects at the N and C termini (Figs. 2 and S2). These extensions may also contribute to more robust PTMs within the CB3 sequences. To ensure straightforward affinity purifications of heterotrimeric assemblies, each chain contained a specific tag placed at the N terminus following the signal peptide (Figure 2, Figure 3 and S2).

We selected the expiCHO transient expression system (Thermo Fisher), which is suitable for our need of coexpression of three different chains (Table S3). The transient transfection system we used allows for rapid production as it eliminates three cycles of time-consuming selection for each vector to generate stable clones and the necessity of each vector having a unique selectable marker. To facilitate collagen-specific PTMs, the expression cell media were supplemented with fresh ascorbate on a daily basis (42, 43).

### Assemblies of eCB3

The chain register of the collagen triple helix has long been considered critical for structure, folding, and function. For collagen IV, the register was explored only for helix 9 (39), and no rules were reported on how it can be translated through the interruptions to other helices. However, in collagen IX, the register of the collagen triple helix N-terminal to the NC2 domain has been defined and shown to be determined by linkage to the NC2 domain (30, 31). This known register allows matching to the correct register of the collagen triple helix if known. As the register of the C-terminal helix (#11, Fig. 1) of eCB3 fragment is not known, we generated plasmids to match each possible register. We refer to an assembly nomenclature in which the His-tagged NC2-A subunit (IX α1) is listed first, the FLAG-tagged NC2-B subunit (IX α2) is listed second, and the Twin-Strep-tagged NC2-C subunit (IX α3) is listed third (Fig. 3). We thus generated 112, 121, and 211 collagen IV eCB3-expressing cells. As it was also not known whether homopolymers assembled and were stable, we also expressed 111 and 222 eCB3 fragments (Fig. 3).

### Purification

**Figure 4:**
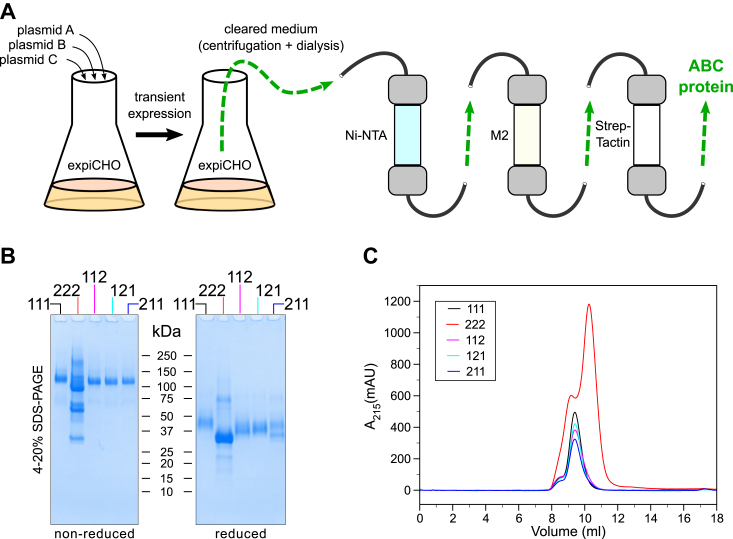
**Production scheme and purification of the extended CB3 (eCB3) assemblies.***A*, three corresponding plasmids A, B, and C were cotransfected and transiently expressed in expiCHO suspension culture. Every day, a fresh ascorbate solution was added to the media to facilitate collagen-specific hydroxylation of proline and lysine residues. The conditioned medium was cleared from cells by centrifugation and extensively dialyzed before a series of affinity purifications. Three affinity columns ensured purification of three differently tagged chains for each eCB3 assembly, that is, 111, 222, 112, 121, and 211. *B*, SDS-PAGE analysis of the purified proteins under nonreduced and reduced conditions. The gel was Coomassie stained. *C*, size-exclusion chromatography profiles of the purified proteins.

Secreted proteins were purified from conditioned cell culture media using three sequential rounds of chain-specific affinity purifications (Figs. S3 and 4*A*). This purification scheme ensures the heterotrimeric composition and high purity (Fig. 4*B*). We achieved yields of pure proteins ranging from 1.5 to 2.4 mg from 1 l of culture medium for 111, 112, 121, and 211 eCB3 assemblies and ∼12 mg for the 222 eCB3 protein.

To determine whether the purified proteins are stabilized by interchain disulfide bonds (Figs. 1*B* and 3), the samples were electrophoresed under both nonreducing and reducing conditions. A single band corresponding to disulfide cross-linked trimer for each of the 111, 112, 121, and 211 eCB3 assemblies was observed at approximately 120 kDa (Fig. 4*B*). The same samples run under reducing conditions resolved in monomeric bands of approximately 40 kDa. This result suggests that register of the last eCB3 helix (#11, Fig. 1), which is deliberately controlled by the NC2 domain, is not critical for assembly. Another surprising observation of assembly of homotrimeric 111 raises a question whether a new isoform of collagen IV can exist. In contrast, the 222-expressing cells produced heterogeneously assembled material with both trimeric, dimeric, and a minority of monomeric-like material under nonreducing conditions. Under reducing conditions, a dominant band at the expected molecular weight for the monomers is observed along with bands of unknown compositions. Because the 222-eCB3 chain is likely poorly or inappropriately folded, we hypothesized it would be sensitive to proteolysis. Indeed, after 6 weeks of storage of purified material at 4 °C, only the 222-eCB3 chain demonstrated significant degradation (Fig. S4).

The affinity-purified proteins were further analyzed by size-exclusion chromatography (Fig. 4*C*). The 111, 112, 121, and 211 eCB3 assemblies demonstrated a single major peak that corresponds to the expected hydrodynamic radius, whereas the major fraction of 222 was shifted to a smaller apparent size suggesting a more globular structure.

### Collagen-specific PTMs

PTM of collagenous sequence is critical for mediating the stability of the collagen helix and collagen function. Our expression system, while not derived from epithelialized cells, should perform the PTMs observed *in vivo* in collagen proteins. We noticed that the 111, 112, 121, and 211 assembly–containing proteins ran as relatively diffused bands on the SDS-PAGE gels. Moreover, the 222-eCB3 chains migrated faster than the other chains under nonreducing and reducing conditions (Fig. 4*B*) suggesting that this protein may not be efficiently post-translationally modified. Consistent with this hypothesis, the amount of secreted and purified 222 was 5 to 8 times higher, as judged from the SDS-PAGE (Fig. 4*B*) and gel filtration chromatography (Fig. 4*C*), possibly because of escaping the PTM machinery by yet unknown mechanism. The 112, 121, and 211 proteins differed from one another in reducing SDS-PAGE (Fig. 4*B*), suggesting that registration differences affected PTMs at chain level. Collectively, these results suggest that the α1-containing eCB3 fragments are post-translationally modified.

**Figure 5:**
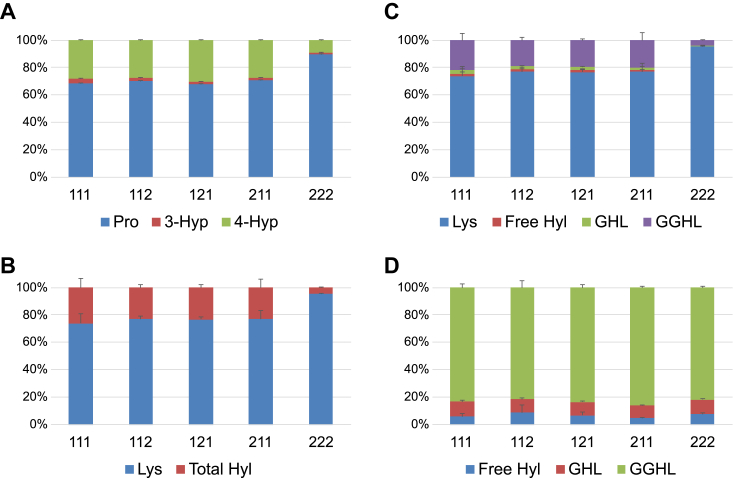
**Collagen-specific post-translational modifications.** The extended CB3 (eCB3) fragments were hydrolyzed and analyzed by LC–MS with stable isotopically labeled collagen used as a reference to quantitate proline (Pro), 4-hydroxyproline (4-Hyp), 3-hydroxyproline (3-Hyp), hydroxylysine (Hyl), galactosyl-hydroxylysine (GHL), and glucosyl-galactosyl-hydroxylysine (GGHL). *A*, overall content of Pro, 4-Hyp, and 3-Hyp. *B*, overall content of Lys and total Hyl after acid hydrolysis, which converted GHL and GGHL into Hyl. *C*, overall content of Lys, Hyl, GHL, and GGHL as a result of a combination of acid hydrolysis (Lys and total Hyl) and base hydrolysis (Hyl, GHL, and GGHL), which does not eliminate sugar moieties. The ratio (Lys + Hyl + GHL + GGHL = 100%) was calculated based on the ratios (Lys + total Hyl = 100%) determined in (*B*) and (total Hyl = Hyl + GHL + GGHL) to compare the two different hydrolysis. *D*, overall content of Hyl, GHL, and GGHL.

To quantitate PTMs of the expressed eCB3 proteins, the amino acid hydrolysates were subjected to LC–MS analysis using stable isotope–labeled collagen (SI-collagen) as a reference (44). Quantities of proline (Pro), 4-hydroxyproline, 3-hydroxyproline, lysine (Lys), hydroxylysine (Hyl), galactosyl-hydroxylysine, and glucosyl-galactosyl-hydroxylysine were calculated as fractions of modified and nonmodified residues (Fig. 5). The 222 assembly stands out as least modified for both Pro and Lys residues, which should destabilize the triple helix and results in a more compact or less elongated structure as shown by elution in gel filtration (Fig. 4*C*). The α1 chain–containing eCB3 assemblies demonstrate approximately equal levels of PTMs. Given that 4-hydroxylation of Pro and hydroxylation and sugar modifications of Lys are restricted to Y position of GXY-tripeptide units, the fraction of modification of these residues reaches ∼50% and ∼35%, respectively.

### Atomic force microscopy imaging

**Figure 6:**
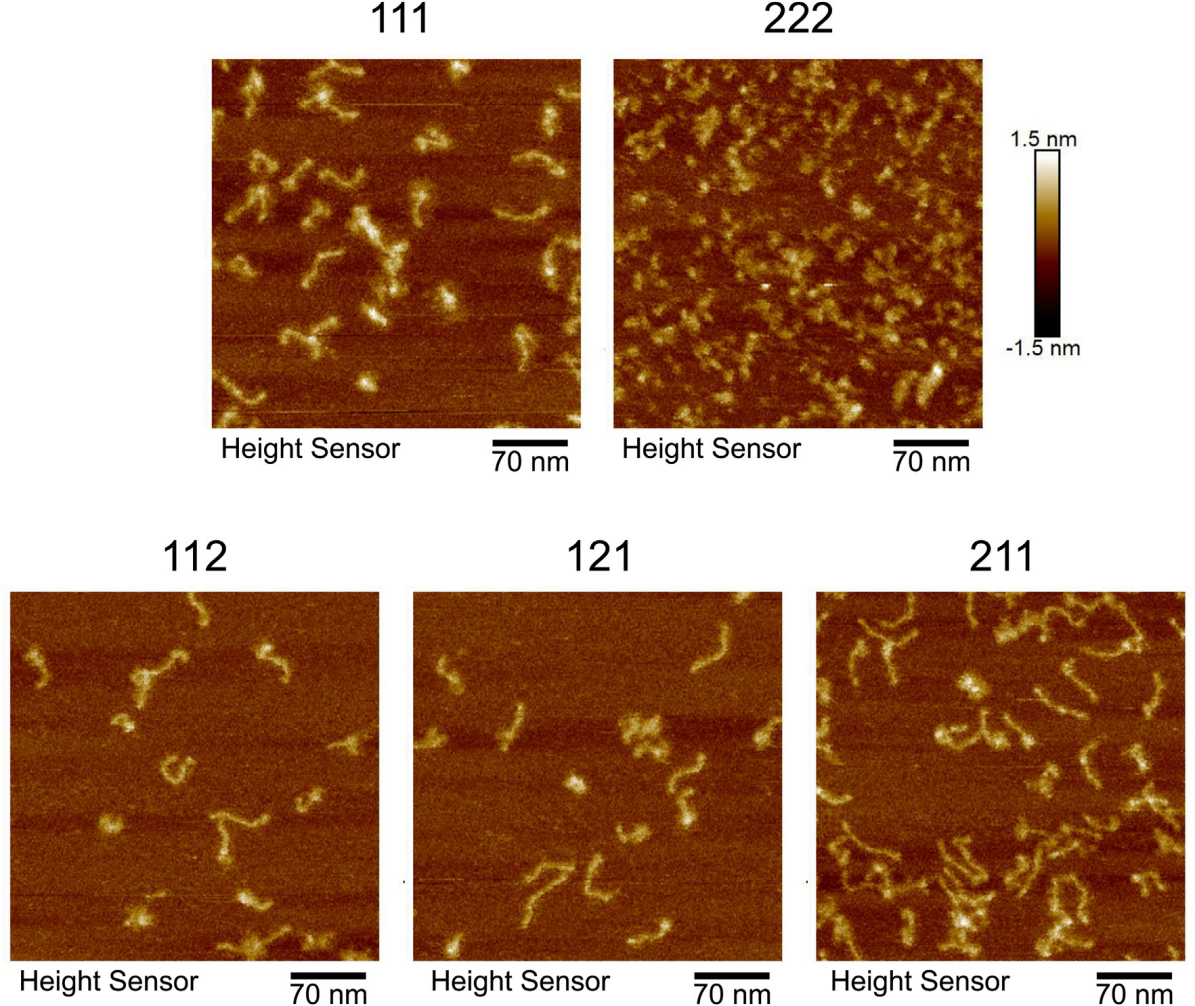
**Atomic force microscopy (AFM) imaging of purified constructs.** Analysis of the extended CB3 (eCB3) assemblies by AFM revealed the presence of individual molecules. All the assemblies except 222 showed individual ∼70-nm-long worm-like structures, each containing a triple helical collagenous domain.

To determine whether the expressed eCB3 proteins were indeed correctly folded into a collagen helix, atomic force microscopy (AFM) imaging was used to reveal the structural organization of individual molecules. All assemblies except 222 eCB3 demonstrated the presence of ∼70-nm-long rod-like structures, which are typical for the triple helical collagenous domain and the α-helical coiled coil domain (NC2 trimer) (Fig. 6). At this resolution, we cannot reliably distinguish triple helical domains from the coiled coil. Some of the molecules showed kinks, possibly at sites of interruptions. No filaments were observed in the 222 eCB3 assembly sample indicating the absence of a folded collagen triple helix.

### CD and thermal transitions

To determine whether the expressed eCB3 assemblies are thermally stable, we analyzed the CD spectra and measured the melting temperature of these proteins. The far UV CD spectra of 111, 112, 121, and 211 assemblies are similar to collagen spectra although without a prominent positive peak in the range of 220 to 230 nm (Fig. 6*A*). The CD spectrum of the NC2 domain alone was previously found predominantly α-helical with a prominent negative peak at 215 to 230 nm (32), which compensates the collagen-specific positive peak in the eCB3 assemblies resulting in a “flat” CD spectrum around 225 nm.

**Figure 7:**
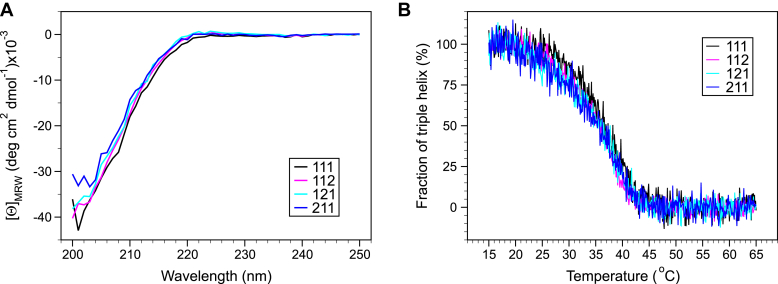
**CD spectra and thermal denaturation of the extended CB3 (eCB3) assemblies.***A*, CD spectra of the eCB3 assemblies in 0.1 M sodium acetate buffer, pH 4.5, at 15 °C demonstrated similar profiles, which indicates similar content of the secondary structure. *B*, thermal unfolding transitions upon heating of the samples were measured at 225 nm and calculated as a fraction of helix. The apparent melting temperature was ∼37 °C for all measured variants. The heating rate was 1 °C/min.

The thermal stability of the complexes was studied at pH 4.5 to prevent disulfide bond reshuffling upon denaturation. At least one unpaired cysteine is expected in 111 eCB3 within the interruption between helices #8 and #9 (Fig. 1). Also, there are no data reported on an oxidative state of the cystine knot. The unfolding transitions of the triple helical domain were measured at 225 nm and calculated as a fraction of helix (Fig. 7). The apparent melting temperature was ∼37 °C for all the assemblies. When both heating and cooling transitions were recorded, they demonstrated a hysteresis phenomenon (Fig. S5) because of slow Pro peptide bond *cis*–*trans* isomerization (45), which causes slow collagen triple helix denaturation and renaturation (46).

### Functional activity of a small-molecule β_1_ integrin inhibitor

The N-(Benzenesulfonyl)-L-prolyl-L-O-(1-pyrrolidinylcarbonyl)tyrosine (BOP) compound was reported to be an inhibitor of α_4_β_1_ and α_9_β_1_ integrins (47), and we determined that this compound inhibits β_1_ integrins, including the collagen-binding integrins (α_1_β_1_, α_2_β_1_, α_10_β_1_, and α_11_β_1_) and α_3_β_1_. Purified α_1_β_1_, α_2_β_1_, α_3_β_1_, α_10_β_1_, and α_11_β_1_ proteins bind to a fluorescently labeled version of the BOP compound as measured by fluorescence polarization (FP). *K*_*d*_ values between the fluorescent BOP compound and α_1_β_1_, α_2_β_1_, α_3_β_1_, α_10_β_1_, and α_11_β_1_ proteins were 13.9, 6.5, 13.4, 16.9, and 30.3 nM, respectively. Using an FP IC_50_ assay with the fluorescent BOP as the FP probe, unlabeled BOP compound has IC_50_ values of 80.7, 16.2, 31.2, 25.3, and 118 nM against α_1_β_1_, α_2_β_1_, α_3_β_1_, α_10_β_1_, and α_11_β_1_, respectively. BOP at a concentration of 50 μM in the solid phase assay is expected to result in near complete inhibition of the integrins’ ability to bind ligands. Thus, small-molecule compound BOP was used as a nonselective integrin inhibitor.

### Integrin binding

**Figure 8:**
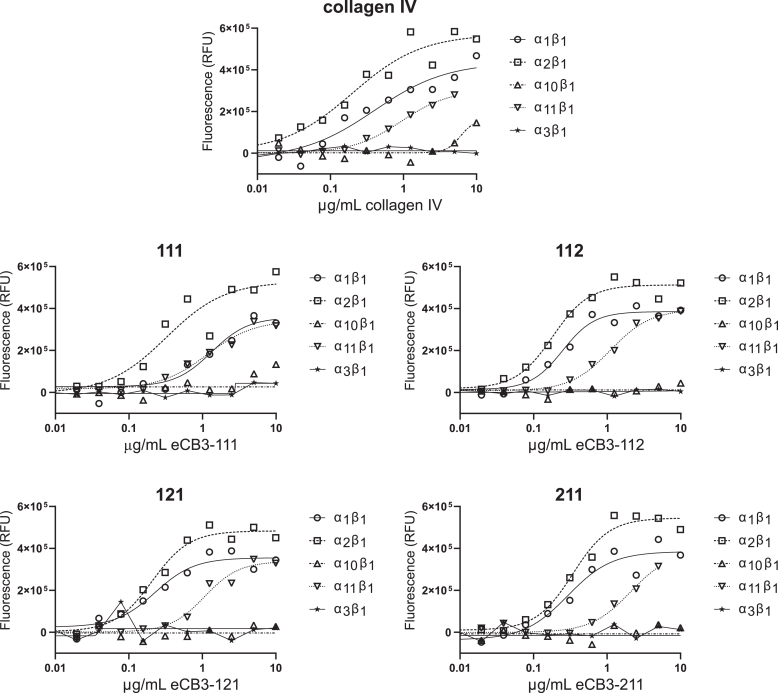
**Binding of integrins α_1_β_1_, α_2_β_1_, α_10_β_1_, α_11_β_1_, and α_3_β_1_ to full-length collagen IV and the extended CB3 (eCB3) assemblies.** Collagen IV and the eCB3 assemblies were coated onto plastic at various concentrations and incubated with various integrins at 5 nM concentration for binding. Bound integrins were detected as fluorescence signal after using the anti-β_1_ biotinylated antibody, which was recognized by peroxidase-coupled streptavidin and fluorogenic peroxidase substrate. Specific binding was calculated as the difference of total and nonspecific binding in the excess of integrin inhibitor BOP.

The solid phase assay was used to measure the binding of the extracellular domains of purified recombinant integrin proteins to collagen IV purified from human tissue and the eCB3 assemblies immobilized onto microtiter plate wells. With immobilized collagen IV purified from human tissue, integrins α_1_β_1_, α_2_β_1_, and α_11_β_1_ bound with saturating binding (Fig. 8), and binding was inhibited with the nonselective small-molecule inhibitor BOP (data not shown). The 111, 112, 121, and 211 eCB3 assemblies bound to integrins α_1_β_1_, α_2_β_1_, and α_11_β_1_ (Fig. 8). No significant binding was observed either for full-length collagen IV or the eCB3 assemblies for the α_3_β_1_ and α_10_β_1_ integrins.

Interestingly, binding to eCB3-111 demonstrated binding to the same integrins as full-length collagen IV suggesting that α2 chain is not absolutely required for integrin interaction.

While integrins were able to bind 111, integrin α_1_β_1_ bound to 112, 121, and 211 with higher apparent affinity as judged by EC_50_ values that were lowered by 5.2-fold, 5.7-fold, and 4.4-fold, respectively (Table 1). This finding emphasizes a role of α2 chain in modulating binding to integrin α_1_β_1_, which was suggested previously as a chain-distributed recognition site within helix #9 (39).

**Table 1:** EC_50_ values of the collagen proteins in solid phase assays with 5 nM purified integrin proteins

Integrin protein	Collagen IV (μg/ml)	eCB3-111 (μg/ml)	eCB3-112 (μg/ml)	eCB3-121 (μg/ml)	eCB3-211 (μg/ml)
α_1_β_1_	0.40	1.33	0.25	0.23	0.29
α_2_β_1_	0.20	0.32	0.18	0.21	0.34
α_3_β_1_	ND	ND	ND	ND	ND
α_10_β_1_	ND	ND	ND	ND	ND
α_11_β_1_	0.93	1.16	1.11	1.07	2.09

Integrin-specific binding was analyzed using four-parameter regression fitting using the GraphPad Prism software.

Abbreviation: ND, not defined.

No other significant differences were noticed for binding of α_2_β_1_ and α_11_β_1_ integrins, suggesting linear nature of binding epitopes within α1 chain of eCB3.

## Discussion

**Figure 9:**
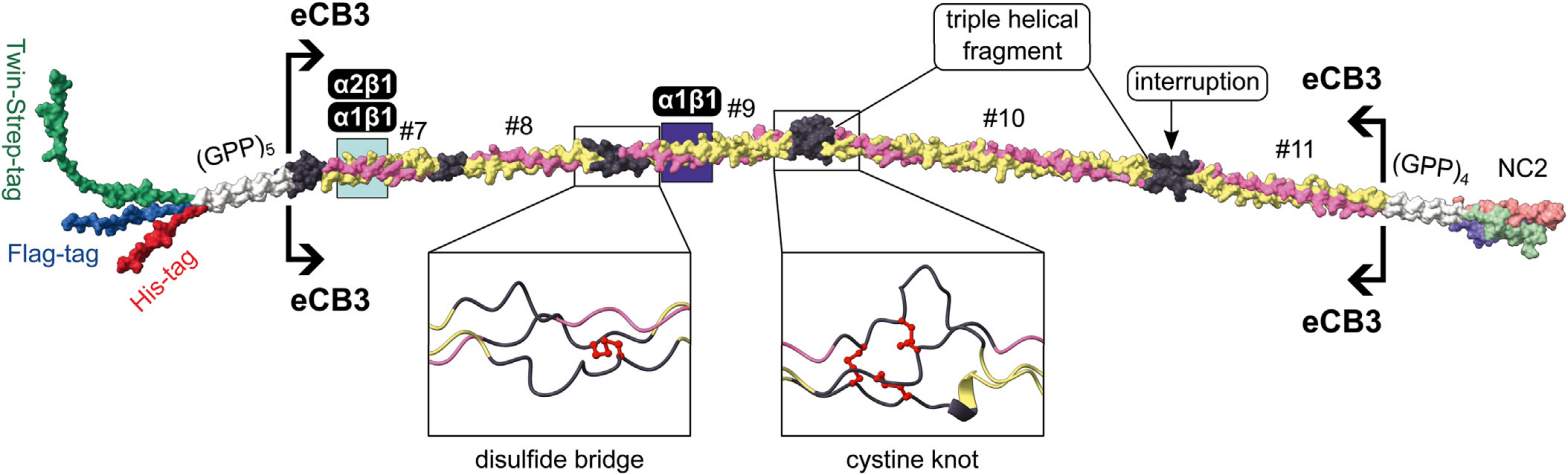
**An overall model of the 112 construct to demonstrate structural organization.** Surface presentation of the model assembled from short overlapping fragments predicted by AlphaFold (69). The model is for demonstration purpose only. Accuracy of the presented model is beyond the scope of this study. The extended CB3 (eCB3) fragment is mapped as follows: the triple helical fragments #7 to 11 are chain colored with *yellow* and *magenta* for α1 and α2, respectively, whereas the interruptions are colored *black*. Other cassette elements are mapped as *white* surface for N- and C-terminal (GPP)_5_ and (GPP)_4_ fragments, the chains of the NC2 domain are colored as *pink*, *light blue*, and *light green*, and tags are colored *red*, *blue*, and *green*. Suggested binding sites for integrins are highlighted by *cyan* and *blue boxes*. *Insets* are demonstrating predicted disulfide bonds within the eCB3 fragment, that is, a disulfide bridge connecting α1 chains of the interruption between helical segments #8 and #9 and three interchain disulfide bonds forming a cystine knot within the interruption between segments #9 and #10.

The insertion of collagenous sequences into a heterotrimerization cassette with the NC2 domain of collagen IX (Fig. 9) allowed recombinant production of well-folded and functional homotrimeric and heterotrimeric collagen fragments. This is the first report of recombinant production of CB3-encompassing region of human collagen IV, which opens the way for investigating the molecular basis of interaction with integrins, heparin, fibronectin, nidogen, perlecan, von Hippel-Lindau protein, HSP47, SPARC (4); collagen IV cleavage by MMP2/9 (4); hereditary angiopathy, nephropathy, aneurysms, and muscle cramps syndrome mutations (35, 48); acute rheumatic fever triggered by *Streptococcus pyogenes* (40). It is now possible to assess effects of point mutations on the binding properties of CB3 and refine studies on shorter fragments.

The outcome of this technology is much broader as it allows us to efficiently produce recombinant fragments of any type of collagen with or without interruptions. Even for homotrimeric types of collagens, our technology allows precise mapping of binding to register-specific residues or studying effects of genetic variants in a composition- and register-specific context. Such fragments could be used to determine whether a genetic variant is pathogenic, a characteristic that is often not obvious from genetic studies. Indeed, among thousands of pathogenic variants reported for collagen IV α1 to α6 chains in the two main variant databases LOVD (https://www.lovd.nl/) and Clinvar (https://www.ncbi.nlm.nih.gov/clinvar/), there are 20 to 70% of variants with conflicting interpretations or uncertain significance (after excluding benign and likely benign variants). Our technology can also be used to explore pathogenic mechanisms, such as the impact of a variant on folding, stability, and interactions with receptors and other macromolecules. It can help to explore an Asp326Tyr variant in α3 chain of collagen IV, which was recently found to be protective against several definitions of diabetic kidney disease (49). Furthermore, the technology is applicable for mapping integrin and other receptor-binding sites to yet unknown positions or fully unexplored collagen isoforms, like α345 of collagen IV.

Interest in trimerization domains as tools to facilitate folding and stabilization of the collagen triple helix started with the use of a foldon domain of bacteriophage T4 fibritin (50) fused to GPP-repeats mimicking the collagen triple helix (51, 52, 53). However, this and other approaches suffered from incompatibility of a threefold rotation symmetry of the trimerization domain and the staggered structure of collagen causing unfavorable kinking at the site of fusion (54). In addition, assembly of heterotrimeric collagen fragments with register control was a challenge until the discovery of the NC2 domain of collagen IX (30, 32). It was successfully used to produce variants of the binding site of collagen I to von Willebrand factor A3 domain in bacteria (30, 31) and collagen-like region of complement component C1q in stably transformed human cells (55).

Here, we developed a general method for cloning and production of a functional region of collagen IV as a framework applicable basically to any fragment of any type of collagen or collagen-like proteins. It allows to control chain composition, chain register, and is applicable to fragments containing irregularities known as triple helix interruptions. It takes advantage of transient coexpression of three chains, which makes it a robust method for production of multiple fragments and/or testing different compositions and chain registers.

Specifically, we produced the CB3-containing fragment of human collagen IV in nonhuman cells. Previously, generation of CB3 fragment was only possible by CNBr cleavage of collagen IV extracted from human tissue. CNBr cleavage is toxic and laborious. Moreover, the sequence analysis revealed that the beginning of α1 chain in CB3 cannot be generated by CNBr as it requires a preceding Met residue. The most frequent natural variant at this position is Pro, and other reported variants, Thr and Ser, are extremely rare, less than 0.00002% according to gnomAD database (56). The closest Met residue is located ∼100 residues upstream (Fig. S6). Thus, generation of CB3 fragment by CNBr cleavage relies also on the yet unknown cleavage mechanism for α1 chain, possibly relying on proteolysis during preparation. With our NC2 recombinant technology, it is now possible to overcome safety issues and generate CB3 and other collagen fragments of desired length and chain composition.

Originally, the NC2 technology involved individual expression of chains in bacteria, assembly of heterotrimers after combining the cell lysates, and subsequent purification of assembled triple helical fragments (30, 31, 32). The approach had several limitations, including the absence of collagen-specific PTMs and solubility issues with certain collagen-derived sequences (unpublished data). The absence of PTMs had several drawbacks including decreased thermal stability of triple helix (lack of stabilizing effect of 4-hydroxylation of Pros) and altered affinity for ligand binding.

These limitations were overcome with the use of a mammalian system and coexpression of all three chains to ensure proper heterotrimer folding inside the cell. Instead of tedious rounds of stable transfections of three compatible plasmids encoding all three chains, we selected transient expression system in Chinese hamster ovary (CHO) cells. The ExpiCHO Expression System is a high-yield transient expression system based on suspension-adapted CHO cells. It uses serum-free medium and is not of human origin, which makes it advantageous for production of proteins for replacement therapy. To ensure high purity and desired chain stoichiometry in the assembled heterotrimer, we used three different chain-specific tags and purified secreted proteins using serially three affinity columns.

The NC2 technology, coupled with expression in CHO cells, allows to combine desired chains in a specific way to obtain different stoichiometries and/or different registers of the chains. In this study, we produced and tested three stoichiometries (α1_3_, α2_3_, and α1_2_α2_1_) and three possible registers for α1_2_α2_1_ composition (α1α1α2, α1α2α1, and α2α1α1). For example, α1α1α2 register means α1 in the leading, α1 in the middle, and α2 in the trailing positions. The register of the chains is influenced by the NC2 domain as it has specific register geometry for the adjacent triple helix (31). We could at least expect the designed register for the last triple helical segment of the collagenous sequence, which is placed in front of NC2. Given the flexible nature of collagen IV interruptions (42, 57, 58, 59), artificial mismatch of the register in the last segment (forced by the NC2 domain) can be tolerated by nearest interruption, and correct (native) register can be restored in the following triple helical segments. Recent success in designing self-assembling collagen-like heterotrimeric peptides with a specific register provides rationale that similar process is possible for native collagen sequences (60, 61, 62). Altogether, it could be an interplay of collagenous and noncollagenous domains in defining the register for the triple helix.

Collagen triple helix is characterized by the presence of unique PTMs, which were found also in our fragments. Indeed, significant PTMs in 111, 112, 121, and 211 eCB3 fragments demonstrate that mammalian system is a better choice for collagen production. Uniquely, 222 fragments revealed least amount of such modifications. Is it just a consequence of overexpression of α2 chains (5–8 more than α1 chains), which overloads the cell PTM machinery, or the presence of α1 chains somehow coordinates the PTM enzymes in a folding triple helix? Those questions need to be further explored. We did not perform analysis of individual sites of PTMs, including glycosylation sites Hyl393 and Hyl543, which were reported to modulate integrin affinity (63). This would be an interesting and important question in future studies.

AFM imaging revealed worm-like structures for all except 222-eCB3 fragments. Their length (∼70 nm) corresponds to the expected lengths of collagenous fragment (66 nm) and NC2 domain (4.4 nm). The collagenous length has been estimated for ∼200 residues of collagen IV and 27 residues of flanking GPP repeats and classical triple helix structure, which gives a rise of 2.9 Å per residue (6). AFM imaging in the air is a straightforward tool for validating collagen triple helical structure. Alternatively, rotary shadowing can be applied if available. Unfortunately, length (∼200 residues per chain) and the presence of multiple interruptions precluded such structural analyses as NMR or X-ray crystallography because of size and increased flexibility, which would otherwise provide atomic resolution details and resolve the chain registers. Nevertheless, this technique has been proven to be successful in solving crystal structures of shorter collagen fragments (31). By generating subfragments of collagen IV CB3 (or other fragments of other collagens), it is possible to elucidate specific integrin-binding sites (or others) and try to determine high-resolution structures.

Thermal transitions of the 111, 112, 121, and 211 eCB3 assemblies demonstrated melting of the triple helical segments at temperatures higher than 25 °C, which is sufficient for *in vitro* experiments at room temperature. If higher thermal stability would be required, as the case for cell culture or animal experiments, a coexpression of collagen prolyl-4-hydroxylase could be an option. Another solution could be use of “collagen-specialized” cultures of cells. HT1080 (human origin) or PF-HR9 (mouse origin) might be a better choice with respect to collagen-specific PTMs, yet several aspects should be taken into consideration like transfection efficiency, nonhuman origin, yield, and interaction with deposited matrix. Further studies are required to broaden repertoire of cell lines suitable for recombinant production of collagen fragments.

Finally, integrin-binding assays confirmed the biological functionality of the eCB3 assemblies. Of the four collagen-specific integrin receptors (64), we observed that binding to three, that is, α_1_β_1_, α_2_β_1_, and α_11_β_1_, integrins demonstrated affinity to collagen IV and the eCB3 assemblies. Under the experimental conditions used, we were not able to confirm binding of α_3_β_1_ and α_10_β_1_ integrins to collagen IV or to the eCB3 assemblies. Here, we reconfirmed the presence of a unique spatially organized binding site to α_1_β_1_ integrin, which requires the presence of α2 chain of collagen IV. Interestingly, α1-homotrimeric variant reveals similar binding efficiency to other two collagen IV–binding integrins, α_2_β_1_ and α_11_β_1_. Taken together, that 111 does form a stable collagen triple helix and binds to integrins, a question is raised whether this combination exists in living tissues, under what conditions and to what extent.

In summary, we developed a system, using collagen IV as a prototype, for recombinant production of triple helical fragments with different combinations of chains and stagger control. We confirmed the structural integrity and stability of the collagenous domain, the collagen-specific PTMs, and functionality at the level of integrin binding. This method opens the door for systematic exploring of the collagen molecules using routine molecular biology techniques and instrumentation.

## Experimental procedures

### DNA constructs

The synthetic genes encoding guest inserts of the eCB3 regions of α1 and α2 chains of human collagen IV and the host frameworks, bearing signal peptides, affinity tags, flanking GPP repeats, and NC2 domain chains of collagen IX, were ordered from Genewiz. The genes were cloned into the pUC-GW-Amp plasmid vector by the supplier (Table S1).

The coding sequences of the host frameworks were excised with HindIII and BspDI restriction enzymes from the cloning plasmids pUC_His-GPP5-2xBsmBI-GPP4-NC2a1, pUC_Flag-GPP5-2xBsmBI-GPP4-NC2a2, and pUC_TwinStrep-GPP5-2xBsmBI-GPP4-NC2a3 (Table S1) and inserted into the backbone of the expression plasmid pRcX (65) using the same restriction sites.

The eCB3 sequences from pUC_a1CB3IV and pUC_a2CB3IV were seamlessly incorporated into the expression vectors pRc_His-GPP5-2xBsmBI-GPP4-NC2a1, pRc_FLAG-GPP5-2xBsmBI-GPP4-NC2a2, and pRc_TwinStrep-GPP5-2xBsmBI-GPP4-NC2a3 using the Golden Gate Assembly BsmBI kit (New England Biolabs, Inc) (Tables S1 and S2).

All plasmids were sequence verified, and their sequences are available upon request.

### Transient expression and purification of proteins

For each assembly, that is, 111, 222, 112, 121, and 211, we transfected three corresponding plasmids (Fig. 2 and Table S3) into expiCHO-S cells (Gibco) according to the ExpiCHO Expression System User Guide and followed the Max Titer Protocol with three modifications: (1) on the day of transfection and every day until the final collection, we supplemented the medium with fresh solution of ascorbic acid to the final concentration of 50 μg/ml; (2) the second feed was added on day 3; and (3) final culture was collected on day 6. The cells were pelleted by centrifugation at 4000*g* for 15 min, and media were collected for protein purification.

The media were extensively dialyzed against Tris-buffered saline (TBS) buffer before three rounds of affinity purifications. First round was purification of His-tagged proteins using nickel–nitrilotriacetic acid resin and the manufacturer protocol (Qiagen). For the second round, the imidazole eluent from round 1 was directly run over the ANTI-FLAG M2 Affinity Gel (Merck KGaA) to capture FLAG-tagged proteins and eluted with FLAG-peptide solution as described (66). Finally, for the third round, the FLAG-peptide elution fraction was loaded onto the Strep-TactinXT Superflow (IBA Lifesciences GmbH) resin to bind Twin-Strep-tagged proteins and eluted with Buffer BXT containing 50 mM biotin as described in the manufacturer protocol for native protein purification.

Finally, we achieved yields of pure proteins ranging from 1.5 to 2.4 mg of 1 l of culture medium for 111, 112, 121, and 211 eCB3 assemblies and ∼12 mg for 222.

### Size-exclusion chromatography

Size-exclusion chromatography was conducted with a Superdex 200 Increase 10/300 GL gel-filtration column (GE Healthcare), using ÄKTA FPLC system (GE Healthcare) at a 0.5 ml/min flow rate. The column was equilibrated with 25 mM Tris–HCl, pH 7.5 supplemented with 150 mM NaCl (TBS). Eluting proteins were monitored by absorbance at 215 nm.

### Quantification of PTMs

Collagen PTMs were analyzed by LC–MS with high sensitivity after amino acid hydrolysis and quantified using SI-collagen as an internal standard. The detailed method is described in the study by Taga *et al.* (44). The molar amounts of Pro, Lys, and their PTMs were calculated from the peak area ratio of the sample (stable isotopically light) relative to the internal standard (stable isotopically heavy), in which molar amounts of each amino acid were predetermined, according to the following formula: (light/heavy) × mol (SI-collagen).

### AFM

The sample preparation for AFM was done on mica (highest grade V1 AFM mica discs, 9.9 mm; Ted Pella). The samples in TBS buffer were diluted 40 times with TBS containing 2 mM CaCl_2_ into ∼2 μg/ml, and 50 μl was deposited onto freshly cleaved mica. After a 30 s incubation period, the excess unbound proteins were washed with ultrapure water for ∼10 s, and the mica was dried immediately under filtered air. All proteins were imaged under dry conditions. AFM imaging was done with a Bruker Dimension Icon atomic force microscope using ScanAsyst/PeakForce mode in air using a SCANASYST-AIR tip.

### CD spectroscopy and thermal unfolding

Far-UV CD spectra were recorded on a Jasco model J-810 spectrometer equipped with Peltier temperature control unit (JASCO Corp) using a quartz cell of 1 mm path length at 20 °C. The proteins were dialyzed against 0.1 M sodium acetate (pH 4.5) buffer, and their final protein concentrations were 0.98, 1.42, 0.86, and 0.93 μM of trimer for constructs 111, 112, 121, and 211, respectively. The spectra were normalized for concentration and path length to obtain the mean molar residue ellipticity. The thermal unfolding transitions were monitored at 225 nm and calculated as fraction of triple helix as described by Bächinger *et al.* (67). The heating rate was 1 °C/min.

### Generation of human α_1_β_1_, α_2_β_1_, α_3_β_1_, α_10_β_1_, α_11_β_1_ integrin ectodomain proteins

Human α_1_ (1–1143), α_2_ (1–1129), and β_1_ (1–728) subunits were cloned separately into the pcDNA3.4 vector. The expression vectors of α_10_β_1_ and α_11_β_1_ were constructed into the pcDNA3.4 backbone as a continuous transcript using the P2A approach. Briefly, the α_10_ (1–1120) or α_11_ (1–1139) subunit was followed by a GGGS linker, and P2A ribosome skipping peptide (ATNFSLLKQAGDVEENPGP), and then the β_1_ subunit. The α subunits contained an ACID coil and StrepII tag in the C terminus; β subunits contained a BASE coil and His tag. All constructs were codon optimized. Expression vectors were transfected to 1 to 4 l of Expi293 cells (Thermo Fisher) and cultured for 3 to 10 days. In most cases, the heterodimeric integrin ectodomain proteins were purified using nickel–nitrilotriacetic acid affinity chromatography, and when necessary, further purified by the StrepTactin affinity column. Finally, the aggregates or monomers were removed from the heterodimer by running a size-exclusion column (Superdex 200 Increase) in the HBS buffer (25 mM Hepes [pH 7.5], 150 mM NaCl, 1 mM MgCl_2_, and 1 mM CaCl_2_). The human α_3_β_1_ protein used in the assays was purchased from R&D Systems (catalog no.: 2840-A3-050).

### FP assays

In the FP *K*_*d*_ assay, dilution series of the purified integrin proteins were incubated in 50 mM Hepes (pH 7.3), 150 mM NaCl, 2 mM Mn^2+^, 0.1 mM Ca^2+^, 0.01% Triton X-100, 1% DMSO, and 3 nM of fluorescent BOP compound (R&D Systems; catalog no.: 6048) (68) in a volume of 20 μl in 384-well plates (PerkinElmer; catalog no.: 6007270), at 22 ⁰C for 1 h. FP was measured with an EnVision plate reader (PerkinElmer) with excitation at 531 nm and emission at 595 nm. *K*_*d*_ values were determined using the total one-site model (GraphPad Prism [Dotmatics]). In FP IC_50_ assays, the integrins at their *K*_*d*_ concentration were incubated in FP assay buffer with a dilution series of the unlabeled BOP compound (R&D Systems; catalog no.: 6047) in a volume of 20 μl in 384-well plates at 22 ⁰C for 1 h. FP was measured, and IC_50_ values were determined using 4-parameter regression fitting.

### Solid phase assays

Human collagen IV was purchased from Advanced Biomatrix (catalog no.: 5022-5MG).

The collagen proteins were diluted in 25 mM Tris–HCl (pH 7.4), 150 mM NaCl, and 2 mM CaCl_2_. About 20 μl of the dilutions were added to wells of a 384-well plate (Corning 3577) and incubated at 4 °C for 16 h. The plates were washed three times with wash buffer consisting of PBS (Cytiva) with 0.05% Tween-20 (Sigma–Aldrich) and blocked with 40 μl of 2.5% bovine serum albumin (Fisher) in PBS at 37 °C for 1 h. The plates were washed three times with wash buffer, and 20 μl of 5 nM integrin protein in 20 mM Hepes (pH 7.3), 150 mM NaCl, 2 mM MnCl_2_, 0.1 mM CaCl_2_, 0.5% bovine serum albumin, 0.01% Triton X-100, 1% DMSO with or without 50 μM BOP inhibitor was added (R&D Systems). The plate was incubated at 22 °C for 1.5 h and washed three times. About 20 μl of the anti-β1 biotinylated antibody (R&D Systems; catalog no.: BAF1778) diluted 1:500 in PBS was added, incubated at 22 °C for 1 h, and washed three times. About 20 μl of the streptavidin–horseradish peroxidase solution (R&D Systems; catalog no.: DY998) was added, incubated at 22 °C for 40 min, and washed five times. About 20 μl of the QuantaBlu working solution (Thermo Fisher) was added, incubated at 22 °C for 15 min, and 20 μl of the QuantaBlu stop solution (Thermo Fisher) was added. Fluorescence (excitation 325 nm and emission 420 nm) was measured on a Cytation 5 plate reader (Agilent). Wells that were not coated with the collagen proteins served as the nonspecific binding control, and the fluorescence value of each coated sample well was subtracted by the average nonspecific binding control value. Next, for each collagen concentration, the fluorescence value of each inhibitor-free sample well was subtracted from the fluorescence value of each BOP-treated sample well to measure integrin-specific binding levels, which were analyzed using 4-parameter regression fitting to calculate EC_50_ values.

### Computer modeling

The amino acid sequences of 112 eCB3 were modeled as six trimeric segments with overlapping. In particular, segment 1 included sequences beginning from the tags to the end of helix #7 (Fig. 1); segment 2: beginning of helix #7 to the end of helix #8; segment 3: beginning of helix #8 to the end of helix #9; segment 4: beginning of helix #9 to the end of helix #10; segment 5: beginning of helix #10 to the end of helix #11; and segment 6: beginning of helix #11 to the end of the molecule (the end of NC2). Each segment was modeled using an open-source code for AlphaFold (DeepMind) (69). Resulting six models were serially superimposed within overlapping sequences, trimmed to remove overlapping residues, and connected to mimic the whole molecule. Finally, the NC2 model was replaced with the crystal structure (Protein Data Bank ID: 5CTD). We observed several mismatches of registers of the modeled triple helical fragments, which ultimately resulted in artificial deletions and insertions. The final model can only be used for demonstration purpose and does not represent a meaningful model.

### Data presentation and analysis

Data were analyzed using the GraphPad Prism and the MS Excel (Microsoft, Inc) software.

Plots were generated and visualized with the MS Excel (Microsoft, Inc) or the Grace program (http://plasma-gate.weizmann.ac.il/Grace/). Protein structure figures were generated using ChimeraX (University of California, San Francisco) (70, 71). Editing and labeling of figures were done using the GIMP (www.gimp.org) and Inkscape (inkscape.org) software packages.

## Data availability

The DNA sequences of the constructs are available on request to S.P.B. and F.Y.L. All other data are contained within the article or Supporting information.

## Supporting information

This article contains supporting information (31, 33, 39, 46, 72).

## Conflict of interest

The authors declare that they have no conflicts of interest with the contents of this article.
