# Supplementary material for: A recombinant technique for mapping functional sites of heterotrimeric collagen helices: Collagen IV CB3 fragment as a prototype for integrin binding
Source: J Biol Chem. 2023 Jun 10;299(7):104901. doi: 10.1016/j.jbc.2023.104901 (PMC10404678; doi:10.1016/j.jbc.2023.104901)
Supplement: Supporting information [file mmc1.docx]

**Supporting Information**

This appendix has been provided by the authors to give readers additional information about their work.

Supplement to: **A recombinant technique for mapping functional sites of hetero-trimeric collagen helices: collagen IV CB3 fragment as a prototype for integrin binding**

***Authors***

Sergei P. Boudko, Elizabeth H. Konopka, Woojin Kim, Yuki Taga, Kazunori Mizuno, Timothy A. Springer, Billy G. Hudson, Terence I. Moy, Fu-Yang Lin

***Content***

Table S1. Plasmids used for cloning and expression S-2

Table S2. Alias names for the expression plasmids S-3

Table S3. Scheme for co-expression of plasmids to produce eCB3 assemblies S-3

Figure S1. CB3 cystine knot conservation throughout the animal kingdom S-4

Figure S2. Schemes and amino acid sequences of encoded polypeptides used
 for production of eCB3 assemblies S-5

Figure S3. Purification of eCB3 fragments S-6

Figure S4. Long-term stability of eCB3 assemblies S-7

Figure S5. Thermal transitions of eCB3 assemblies measured by CD S-8

Figure S6. Putative cyanogen bromide cleavage sites in human collagen IV S-9

References S-9

Table S1. **Plasmids used for cloning and expression**.

| **Plasmid name** | **Content** | **Type** |
| --- | --- | --- |
| pUC_His-GPP5-2xBsmBI-GPP4-NC2a1 | Host framework with His-tag and α1 chain of NC2 domain of human collagen IX | cloning |
| pUC_Flag-GPP5-2xBsmBI-GPP4-NC2a2 | Host framework with His-tag and α2 chain of NC2 domain of human collagen IX | cloning |
| pUC_2xStrep-GPP5-2xBsmBI-GPP4-NC2a3 | Host framework with His-tag and α3 chain of NC2 domain of human collagen IX | cloning |
| pRcX | Expression vector with CMV promoter | expression |
| pRc_His-GPP5-2xBsmBI-GPP4-NC2a1 | Host framework with His-tag and α1 chain of NC2 domain of human collagen IX | cloning/expression |
| pRc_Flag-GPP5-2xBsmBI-GPP4-NC2a2 | Host framework with His-tag and α2 chain of NC2 domain of human collagen IX | cloning/expression |
| pRc_2xStrep-GPP5-2xBsmBI-GPP4-NC2a3 | Host framework with His-tag and α3 chain of NC2 domain of human collagen IX | cloning/expression |
| pUC_a1CB3IV | CB3 extended sequence of α1 chain of human collagen IV | cloning |
| pUC_a2CB3IV | CB3 extended sequence of α2 chain of human collagen IV | cloning |
| pRc_His-GPP5-CB3a1-GPP4-NC2a1 | CB3 α1 guest sequence in the NC2 α1 host, His-tagged | expression |
| pRc_His-GPP5-CB3a2-GPP4-NC2a1 | CB3 α2 guest sequence in the NC2 α1 host, His-tagged | expression |
| pRc_Flag-GPP5-CB3a1-GPP4-NC2a2 | CB3 α1 guest sequence in the NC2 α2 host, FLAG®-tagged | expression |
| pRc_Flag-GPP5-CB3a2-GPP4-NC2a2 | CB3 α2 guest sequence in the NC2 α2 host, FLAG®-tagged | expression |
| pRc_2xStrep-GPP5-CB3a1-GPP4-NC2a3 | CB3 α1 guest sequence in the NC2 α3host, Twin-Strep®-tagged | expression |
| pRc_2xStrep-GPP5-CB3a2-GPP4-NC2a3 | CB3 α2 guest sequence in the NC2 α3 host, Twin-Strep®-tagged | expression |

Table S2. **Alias names for the expression plasmids**.

| **Plasmid name** | **Alias** |
| --- | --- |
| pRc_His-GPP5-CB3a1-GPP4-NC2a1 | 1-1 |
| pRc_His-GPP5-CB3a2-GPP4-NC2a1 | 2-1 |
| pRc_Flag-GPP5-CB3a1-GPP4-NC2a2 | 1-2 |
| pRc_Flag-GPP5-CB3a2-GPP4-NC2a2 | 2-2 |
| pRc_2xStrep-GPP5-CB3a1-GPP4-NC2a3 | 1-3 |
| pRc_2xStrep-GPP5-CB3a2-GPP4-NC2a3 | 2-3 |

Table S3. **Scheme for co-expression of plasmids to produce eCB3 assemblies**. The register is defined following the BAC-rule, *i.e.* chain B in the leading position, A in the middle, and C in the trailing (1).

| **eCB3 assembly** | **Plasmids co-transfected**  (see Table S2 or Fig. 3 for reference) | **Anticipated register of the last helical segment** |
| --- | --- | --- |
| 111 | 1-1  1-2  1-3 | α1α1α1 |
| 222 | 2-1  2-2  2-3 | α2α2α2 |
| 112 | 1-1  1-2  2-3 | α1α1α2 |
| 121 | 1-1  2-2  1-3 | α2α1α1 |
| 211 | 2-1  1-2  1-3 | α1α2α1 |


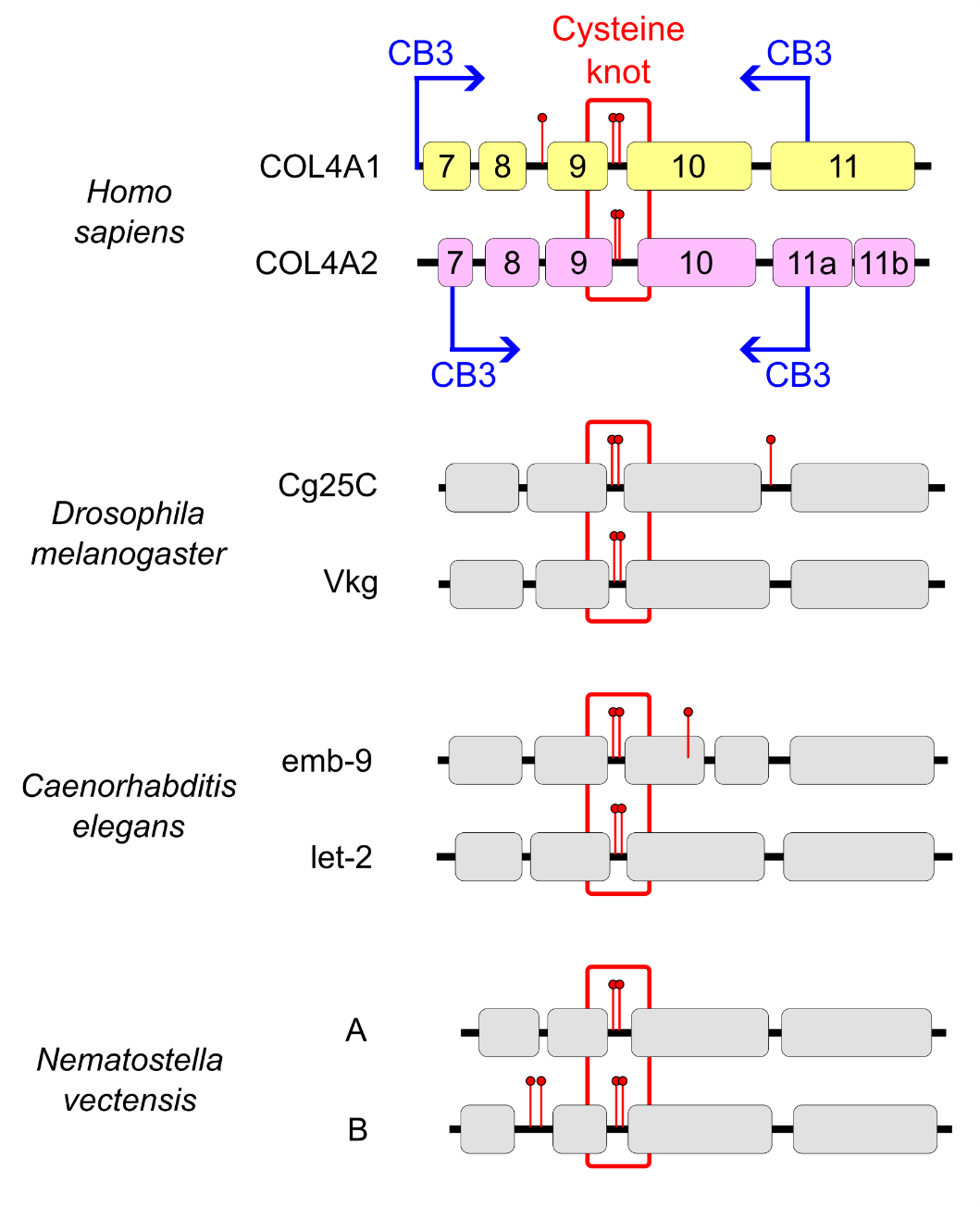


Figure S1. **CB3 cystine knot conservation throughout the animal kingdom**. The cystine knot was recognized within the CB3 fragment of human collagen IV (2). It is presumably formed by interchain disulfides between pairs of cysteines in α1 and α2 chains within the interruption connecting helical segments 9 and 10. Although there is variability of helical segments, interruptions, and cysteines within the CB3-corresponding region across the animal species, pairs of cysteines in α1 and α2 chains from human to cnidarians are conserved, which suggests also evolutionary conservation of the cystine knot. Cysteines are depicted as red pins. Those cysteines forming the CB3 cystine knot are highlighted by red underlying boxes. Uniprot identifiers for sequences are: P02462 for human COL4A1 (isoform 1) and P08572 for human COL4A2; P08120 for Cg25C and Q9VMV5 for Vkg of *D. melanogaster*; P17139 for emb-9 and P17140 for let-2 of *C. elegans*; V9GW22 for chain A of *N. vectensis*. The sequence for chain B of *N. vectensis* was assembled from an open reading frame of the genome (will be published elsewhere).

Figure S2. **Schemes and amino acid sequences of encoded polypeptides used for production of eCB3 assemblies.** Each sequence begins with the signal peptide followed by a specific tag sequence, i.e. His tag for chain A, Flag tag for chain B, and TwinStrep for chain C. The collagenous sequence (eCB3) is flanked by five and four GPP tripeptide units for avoiding side effects. Each construct ends with a unique chain of the NC2 domain of collagen IX (α1 in chain A, α2 in chain B, and α3 in chain C), which is responsible for chain selection and registering. In cursive are sequences not found in original CB3 fragments (3).


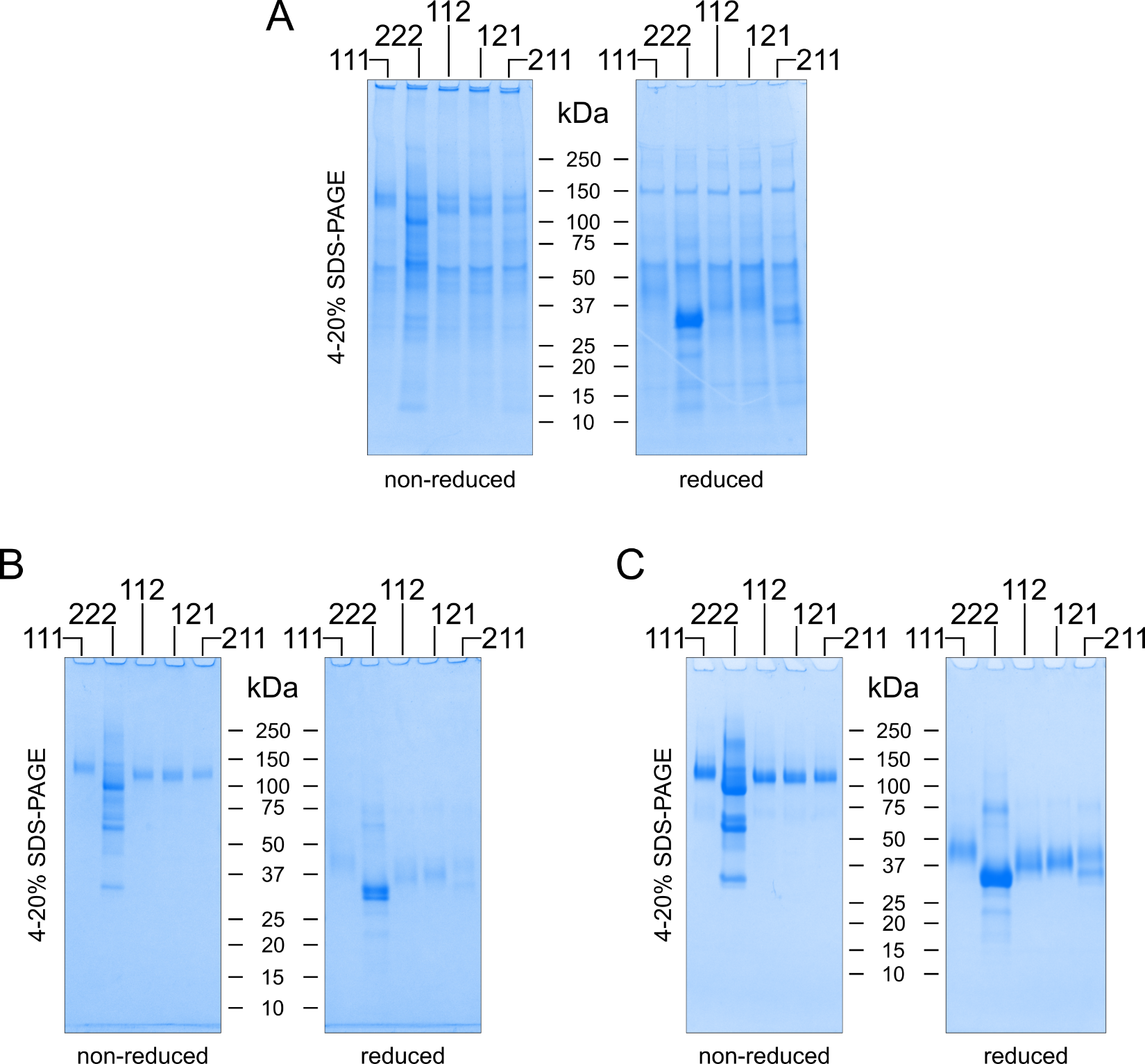


Figure S3. **Purification of eCB3 fragments**. The proteins were purified serially over the three columns. Eluates after each type of column were analyzed on the SDS-PAGE with Coomassie staining. (**A**) Ni-NTA column for His-tagged proteins. (**B**) M2 agarose column for Flag-tagged proteins. (**C**) Strep-Tactin column for Twin-Strep-tagged proteins. Panel (**C**) is reused from Fig. 4B for comparison purpose.


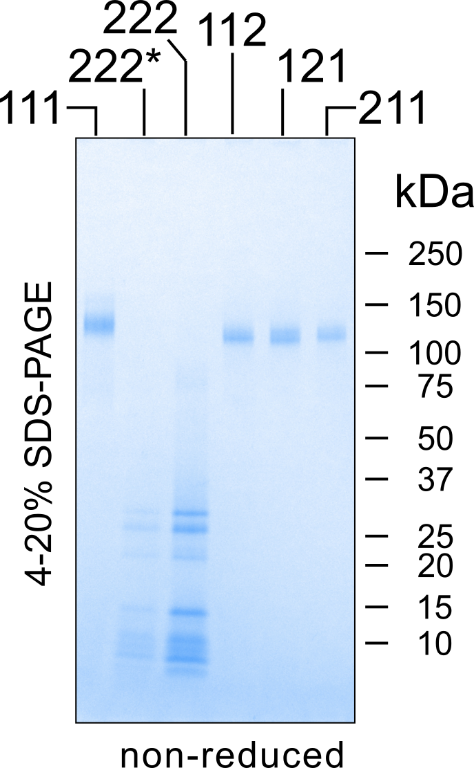


Figure S4. **Long-term stability of eCB3 assemblies.** The major peak for each sample was pooled after the size-exclusion chromatography, stored at 4 °C for 20 weeks, and re-analyzed on the SDS-PAGE with Coomassie staining. 222* - analysis of an additional sample pooled from the left shoulder of the major peak of 222. Only 222 samples demonstrated significant degradation.


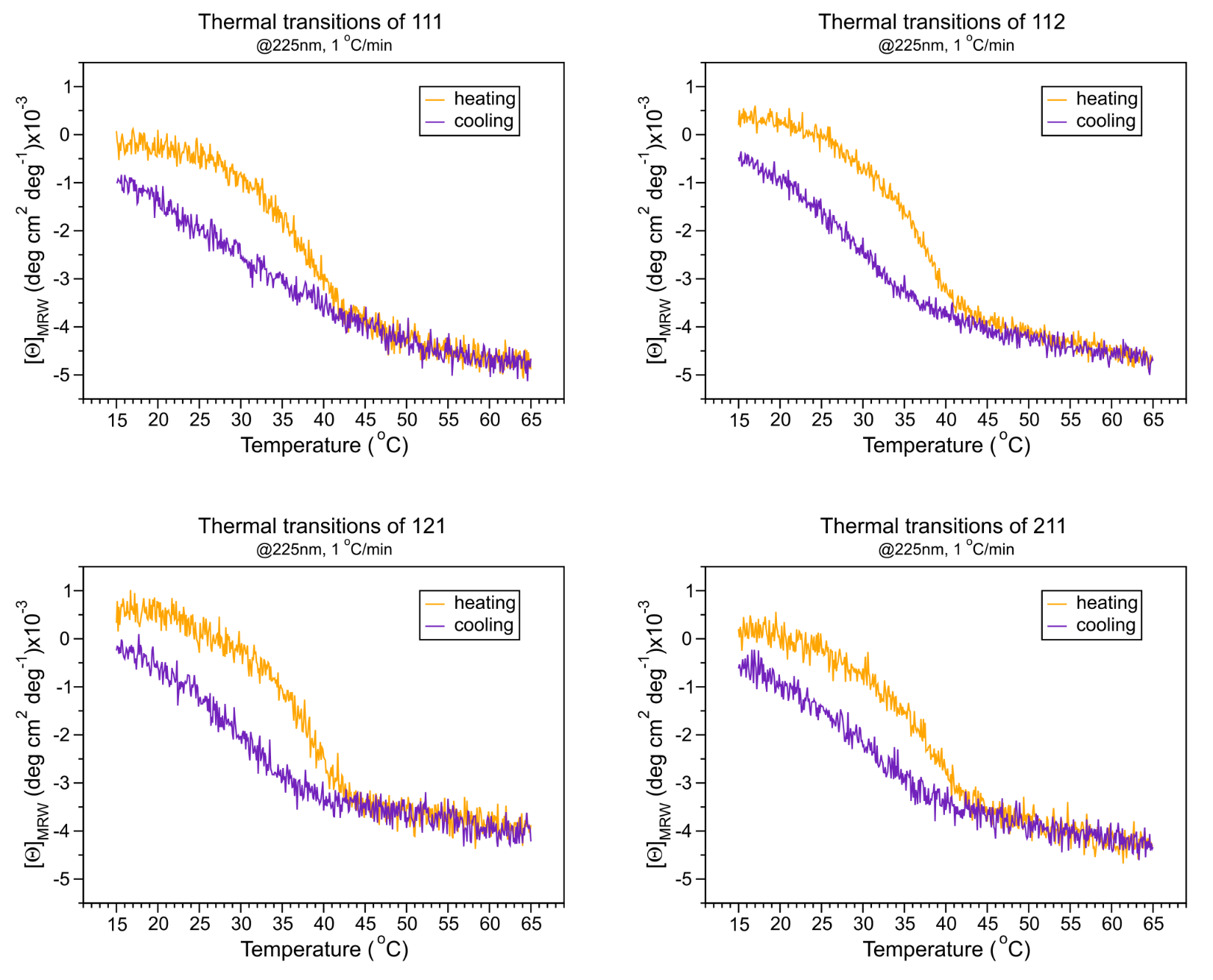


Figure S5. **Thermal transitions of eCB3 assemblies measured by CD.** Mean molar ellipticity is shown at 225 nm wavelength. Heating (orange) and cooling (purple) curves were measured at 1 °C/min rate. All assemblies demonstrated pronounced lag in gaining back the secondary structure signal upon cooling, a well-known phenomenon of collagen hysteresis (4,5).

Figure S6. **Putative cyanogen bromide cleavage sites in human collagen IV.** Reported sequences for CB3 fragment span residues G371-M554 for α1 chain and G407-M570 for α2 chain (3). In case of α1 chain the expected residue for specific cyanogen bromide cleavage in position 370 should be M, but it is P (shown in red). The nearest possible cleavage site is M271, which is located ~100 residues upstream. The cleavage at P370 was possibly non-specific during CB3 preparation. The Uniprot identifiers for sequences are: P02462 for α1 (isoform 1) and P08572 for α2.

***References***

1. Boudko, S. P., and Bachinger, H. P. (2016) Structural insight for chain selection and stagger control in collagen. *Sci Rep* **6**, 37831

2. Vandenberg, P., Kern, A., Ries, A., Luckenbill-Edds, L., Mann, K., and Kuhn, K. (1991) Characterization of a type IV collagen major cell binding site with affinity to the alpha 1 beta 1 and the alpha 2 beta 1 integrins. *J Cell Biol* **113**, 1475-1483

3. Eble, J. A., Golbik, R., Mann, K., and Kuhn, K. (1993) The alpha 1 beta 1 integrin recognition site of the basement membrane collagen molecule [alpha 1(IV)]2 alpha 2(IV). *EMBO J* **12**, 4795-4802

4. Engel, J., and Bachinger, H. P. (2000) Cooperative equilibrium transitions coupled with a slow annealing step explain the sharpness and hysteresis of collagen folding. *Matrix Biol* **19**, 235-244

5. Mizuno, K., Boudko, S. P., Engel, J., and Bachinger, H. P. (2010) Kinetic hysteresis in collagen folding. *Biophys J* **98**, 3004-3014
